# Chloroquine and COVID-19—A systems biology model uncovers the drug’s detrimental effect on autophagy and explains its failure

**DOI:** 10.1371/journal.pone.0266337

**Published:** 2022-04-07

**Authors:** Orsolya Kapuy, Tamás Korcsmáros

**Affiliations:** 1 Semmelweis University, Department of Molecular Biology at the Institute of Biochemistry and Molecular Biology, Budapest, Hungary; 2 Earlham Institute, Norwich Research Park, Norwich, United Kingdom; 3 Quadram Institute Bioscience, Norwich Research Park, Norwich, United Kingdom; Faculty of Medicine, University of Belgrade, SERBIA

## Abstract

The COVID-19 pandemic caused by SARS-CoV-2 has resulted in an urgent need for identifying potential therapeutic drugs. In the first half of 2020 tropic antimalarial drugs, such as chloroquine (CQ) or hydroxochloroquine (HCQ) were the focus of tremendous public attention. In the initial periods of the pandemic, many scientific results pointed out that CQ/HCQ could be very effective for patients with severe COVID. While CQ and HCQ have successfully been used against several diseases (such as malaria, autoimmune disease and rheumatic illnesses); long term use of these agents are associated with serious adverse effects (i.e. inducing acute kidney injury, among many others) due to their role in blocking autophagy-dependent self-degradation. Recent experimental and clinical trial data also confirmed that there is no sufficient evidence about the efficient usage of CQ/HCQ against COVID-19. By using systems biology techniques, here we show that the cellular effect of CQ/HCQ on autophagy during endoplasmic reticulum (ER) stress or following SARS-CoV-2 infection results in upregulation of ER stress. By presenting a simple mathematical model, we claim that although CQ/HCQ might be able to ameliorate virus infection, the permanent inhibition of autophagy by CQ/HCQ has serious negative effects on the cell. Since CQ/HCQ promotes apoptotic cell death, here we confirm that addition of CQ/HCQ cannot be really effective even in severe cases. Only a transient treatment seemed to be able to avoid apoptotic cell death, but this type of therapy could not limit virus replication in the infected host. The presented theoretical analysis clearly points out the utility and applicability of systems biology modelling to test the cellular effect of a drug targeting key major processes, such as autophagy and apoptosis. Applying these approaches could decrease the cost of pre-clinical studies and facilitate the selection of promising clinical trials in a timely fashion.

## Introduction

The recent coronavirus infection related disease (COVID-19) has rapidly became a global pandemic by March 2020 [1, 2]. Due to the severity of the pandemic, there was an urgent need for finding effective treatments using medicines already on the market [3, 4]. Alongside the development of vaccines against COVID-19, there have been intensive attempts to discover effective drugs that slow down the spread of disease or decrease its severity [5, 6].

COVID-19 is caused by the severe acute respiratory syndrome coronavirus 2 (SARS-CoV-2) [7]. The illness might be mild or severe, often depending on the consequence of age associated diseases or other chronic diseases (i.e. cardiovascular pulmonary, diabetes or immune compromised conditions) [8, 9]. COVID-19 patients with more severe disease have also been found to experience a so-called cytokine storm leading to severe lung damage with fatal consequences [10, 11].

The molecular mechanism of coronavirus infection has also been studied on the cellular level [12, 13]. It has already shown that replication of SARS-CoV-2 induces endoplasmic reticulum (ER) stress by severely restructuring the ER membrane [14, 15]. The ER is used to form double membrane vesicles for viral genome replication during SARS-CoV-2 infection, which results in activation of an ER stress response mechanism, called the unfolded protein response (UPR) [16, 17]. UPR always induces autophagy-dependent survival, but severe ER stress results in apoptotic cell death [18, 19]. It has been reported that autophagy fights successfully against “mild” coronavirus infection by digesting viral components [16, 20, 21].

Cellular autophagy occurs at baseline level of activity during physiological conditions, where it plays crucial role in removing and recycling effete organelles and proteins by sequestering them into a vesicle, called autophagosome and later degraded by the enzymes within lysosomes [22, 23]. Autophagy can also be included in times of cellular starvation or stress (e.g. secondary to genotoxic or biochemical stress or infection) as a cellular survival mechanism [22, 24]. Thus, the activity of autophagy in cells is tightly regulated. It is well-known that the key activator of autophagy is ULK1/2 (unc51-like autophagy activating kinase 1/2), the mammalian homolog of yeast Atg1 [25, 26]. ULK1/2 controls the early stage of autophagy forming a so-called autophagy induction complex [27–30]. ULK1/2 can phosphorylate Beclin1 (the mammalian homolog of yeast Atg6), thereby promoting to activate the class III phosphatidyl inositol-3-kinase [31]. Neither Beclin1 nor ULK1 depleted cells can induce the autophagy [23, 27, 32–34].

It is well-established that intolerable levels of SARS-CoV-2 viruses in the cell disrupts this cellular survival process by usurping the components of the autophagy pathway, leading to apoptotic cell death of the host cell [35].

Antimalarial drugs such as chloroquine, and its less toxic derivative, hydroxychloroquine (CQ/HCQ) were quickly suggested as a possible therapeutic option for COVID-19 during the initial stages of the pandemic [6, 36–38]. Previously, CQ/HCQ treatment has been an important therapeutic option for several autoimmune diseases, and this treatment is one of the safest immunomodulatory agent for rheumatic illness [39, 40]. Both drugs have a long half-life and a well-characterised mechanism of action, and they are well-known inhibitors of autophagy [41, 42]. CQ/HCQ gets accumulated in the lysosomes and inhibits the lysosome-mediated cellular proteolysis and the formation of autolysosomes by increasing the endosomal/lysosomal pH [43].

Although CQ/HCQ seemed to be safe for the treatment of COVID-19 initially, sometimes serious adverse effects have been reported, mostly with long term use [37, 44]. It was also observed that CQ/HCQ treatment was not suitable for patients with conditions such as diabetes, hypertension and cardiac issues [45, 46]. At present, an abundance of research articles has focused on the controversial role of CQ/HCQ in COVID-19 treatment with regards to their clinical safety and efficacy [6], but many scientific questions about their effects have not yet been answered.

To address systems-level questions on drug effectiveness, biologists often apply mathematical models to understand the precise molecular mechanisms that control important aspects of cell physiology [47, 48]. These models also help us to understand the dynamic characteristics of certain treatments and drugs in human cells [49, 50]. Although the complexity of cellular systems makes this analysis highly challenging, a reductionist approach using a simple wiring diagram can be created containing the key components of the control network to address fundamental questions [47, 49]. Accordingly, we have focused our research on the systems biology analysis of autophagy and its crosstalk with apoptosis with respect to ER stress [51–53]. Recently, we have shown by using both molecular and systems biology methods that autophagy always generates an evidently distinguishable threshold for apoptosis activation upon ER stress [51, 53]. Our four-component model (i.e. two ER stress sensors, autophagy and apoptosis inducers) confirmed the importance of autophagy-dependent survival [18]. Here, we introduce a mathematical model adapting our previously published results about cellular life-and-death decision with respect to ER stress. In this report, we claim that mild COVID-19 induced cellular stress, similar to ER stress, can be readily surmounted by the host cell via autophagy However, severe SARS-CoV-2 infection completely disrupts autophagy and promotes apoptotic cell death. We also show that long-term CQ/HCQ treatment has adverse effects at the cellular level by inducing apoptotic cell death. In this report we demonstrate that a systems biology approach to the theoretical analysis of the dynamic characteristics of a drug of interest (i.e. CQ/HCQ here) could be very important in biomedical and clinical research and support pharmacological developments.

## Materials and methods

**Fig 1:**
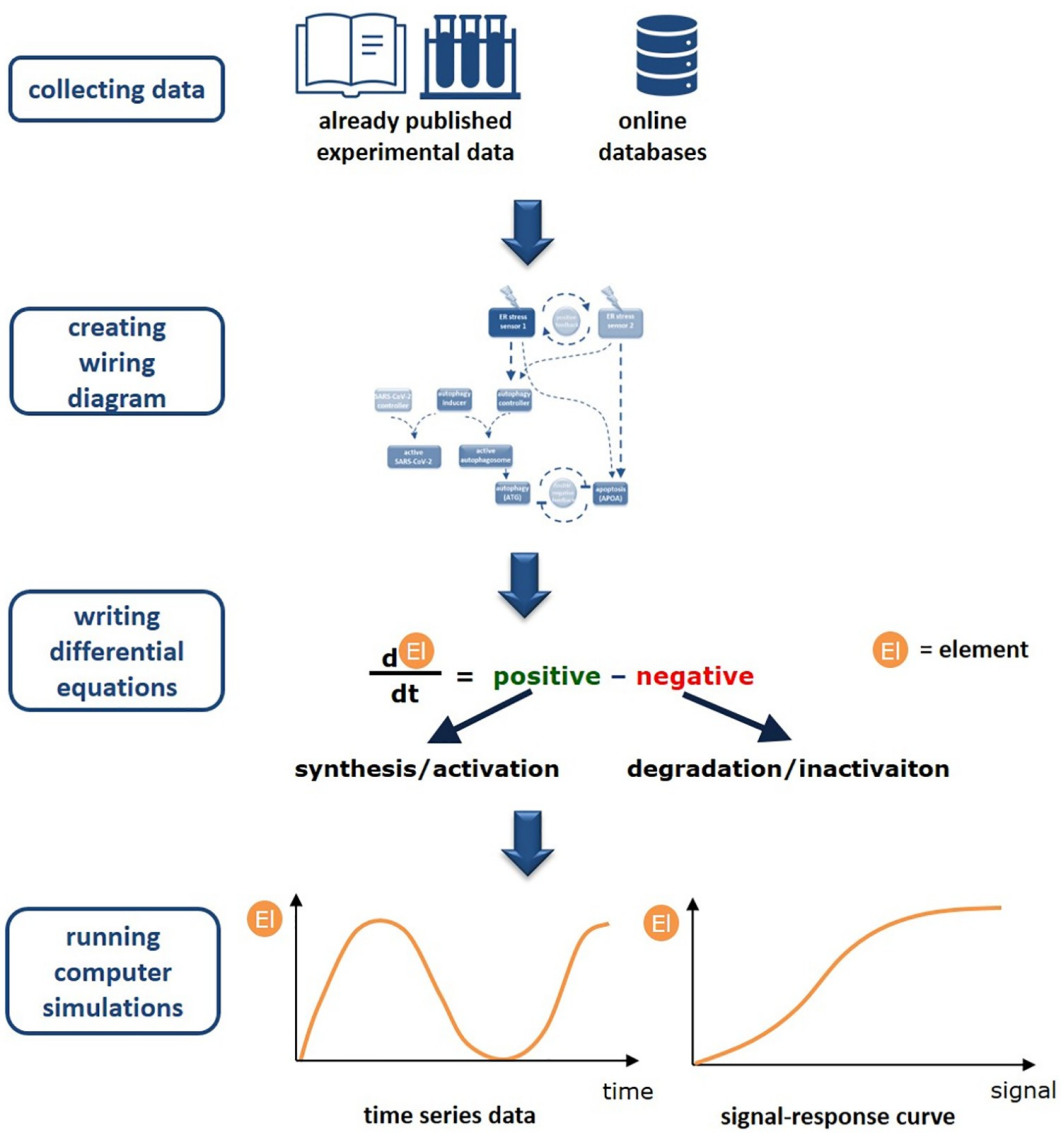
The schematic picture of our workflow. The modelling approach we present here is based on existing experimental data, either from the published papers or from databases containing referenced information. We use this data to create a static wiring diagram and differential equitations to capture the dynamics of the system. The models are then used to simulate the effect of the different elements ("El" for short).

A systems-level view can be developed by bringing together the components and interactions reported in the literature. The schematic description about building up our mathematical model is seen on Fig 1. Our wiring diagrams are independent of identity of molecular players including only the relevant systems-level feedback loops.

Such a network can be translated into a set of mathematical equations that describe how each component concentration/activity in the network changes with the time. The rate of change of a component is described by ordinary differential equation (ODE) based on biochemical reaction kinetics. Each biochemical reaction is represented as a term on the right hand side of the ODE for a component participating in the reaction [54, 55]. Each reaction in the network can be described either by using law of mass action or Michaelis-Menten kinetics [56–58].

With this mathematical modelling the qualitative features of a dynamic system can be easily explored by using both computational simulations and signal-response curves (for more description about the modelling see S1 Text in S1 File).

In this work, the temporal profiles and signal response curves were computed numerically using *XPP-AUT*. All the simulations presented in the text are based on XPP codes found in S2 Text in S1 File. The starting parameter set was able to refer to physiological conditions. The parameters values were perturbed to capture all the possible qualitative behaviours that the given network can exhibit.

## Results and discussion

### A control network models the CQ/HCQ treatment related inhibition of autophagy regulation with respect to ER stress

**Fig 2:**
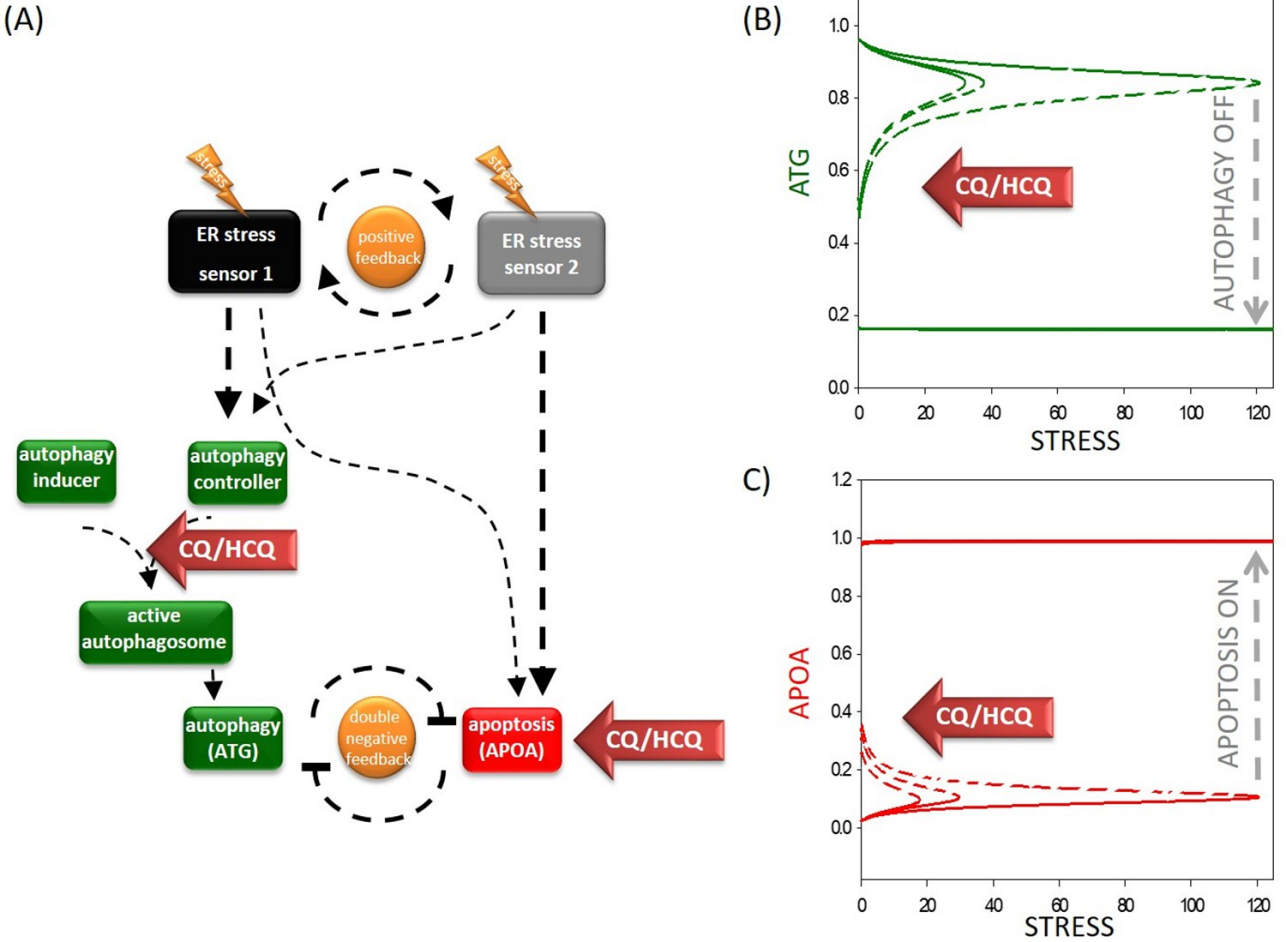
The simple network of autophagy-dependent survival upon chloroquine treatment during cellular stress. **(A) The wiring diagram of the response mechanism**. The active form of the ER stress sensor 1 and 2, autophagy inducer, autophagy controller, active autophagosome, ATG and APOA are grouped together in isolated coloured boxes. ATG represents the active form of autophagy activator complex, while APOA represents the active form of apoptosis. Dashed lines show how the molecules can influence each other. Blocked end lines denote inhibition. Red arrows labelled with “chloroquine” show where the drug treatment affects the control network, while yellow “thunders” symbolize the cellular stress. **The signal-response curves of (B) survival process and (C) cell death process are shown with respect to increasing stress level**. On the “x” axis the stress level, while on the “y” axis the relative activity of (B) ATG or (C) APOA are plotted. Solid lines denote stable state, while dashed line denotes the unstable state of the control network at a given stress level. Grey dashed arrows depict the threshold for autophagy inactivation / cell death activation. Red arrows labelled with “chloroquine” show how the signal-response curves are changing according to increasing level of chloroquine (for the code, see S1 File).

We created mathematical models to understand the dynamic characteristic of the regulatory network of the ER cellular stress response mechanism. Although we know that there are three sensors of the ER stress induced signal transduction pathway, for simplicity, in this analysis, according to our previous model [18, 51], we claim that ER stress is induced *via* two ER stress sensors (see Fig 1A). The two ER stress sensors help each other through a positive feedback loop. While one sensor more powerfully activates autophagy-dependent survival, the other is more supportive of apoptosis. However, to better understand the dynamic behaviour of the autophagy-dependent survival mechanism following CQ/HCQ treatment, here we also considered the steps of autophagosome formation. In our reductionist model we state that ER stress sensors promote a so-called autophagy controller (this includes all the cellular elements which can induce the stress response mechanism, such as Beclin1, Atg14, Vps34). This autophagy controller is able to form an active autophagosome by making a complex with an autophagy inducer. This autophagy inducer includes all the components that are essential for the autophagosome formation but not directly activated by the stress response mechanism, i.e. ATG12 and LC3. The active autophagosome can induce the survival process in our model via a so-called autophagy effector (see Fig 2A). For simplicity we assume that this apoptosis effector includes all the components which are crucial for a fatal decision (for details about the references of the regulatory connections and codes and software used for simulations see the Materials and Methods and S1-S2 Text, S1 Table in S1 File).

To test the accuracy of our model, first the signal-response curves of both autophagy and apoptosis effectors are plotted (Fig 2B and 2C). The signal-response curve shows how autophagy and apoptosis become active with respect to ER stress. Corresponding with our previous data, we confirm here that autophagy gets activated even at low levels of ER stress to promote the survival process. However, apoptosis remains inactive due to the double negative feedback loop between them. If the stress level reaches a critical threshold, cell death gets rapidly activated and down-regulates the transient autophagy-dependent survival due to the toggle switch between the two processes (see the grey dashed arrows on Fig 2B and 2C). It is well-known that apoptosis induction is irreversible, namely apoptosis cannot be switched on later, even if the cellular stress is removed.

To explore the effect of CQ/HCQ treatment, corresponding to already published data we first had to decide that exactly at which step does CQ/HCQ exert its effect on autophagy in our model. Based on experimental data we claim that CQ/HCQ disrupts the autophagosome formation [59]. Therefore, a functional complex formation between the autophagy controller and autophagy inducer were diminished following addition of CQ/HCQ by reducing the total amount of autophagy inducer in the model. Besides, CQ/HCQ can induce apoptotic cell death in the model referring to the autophagy-independent biological effects of the drug [60]. In this case, drastic changes could be observed on the signal-response curves (see the red arrows labelled with “CQ/HCQ” on Fig 2B and 2C). Namely, the thresholds on both curves move to the left which results in autophagy turnings off at a much lower threshold, meanwhile cell death gets activated. These results correspond with those results which claim that CQ/HCQ have a drastic negative effect on autophagy, and it was also previously shown experimentally that the drug induces apoptosis [59, 61].

Our results clearly confirm that CQ/HCQ have a negative effect on autophagy-dependent survival with respect to ER stress.

### SARS-CoV-2 influences autophagy-dependent survival depending on the level of the infection

**Fig 3:**
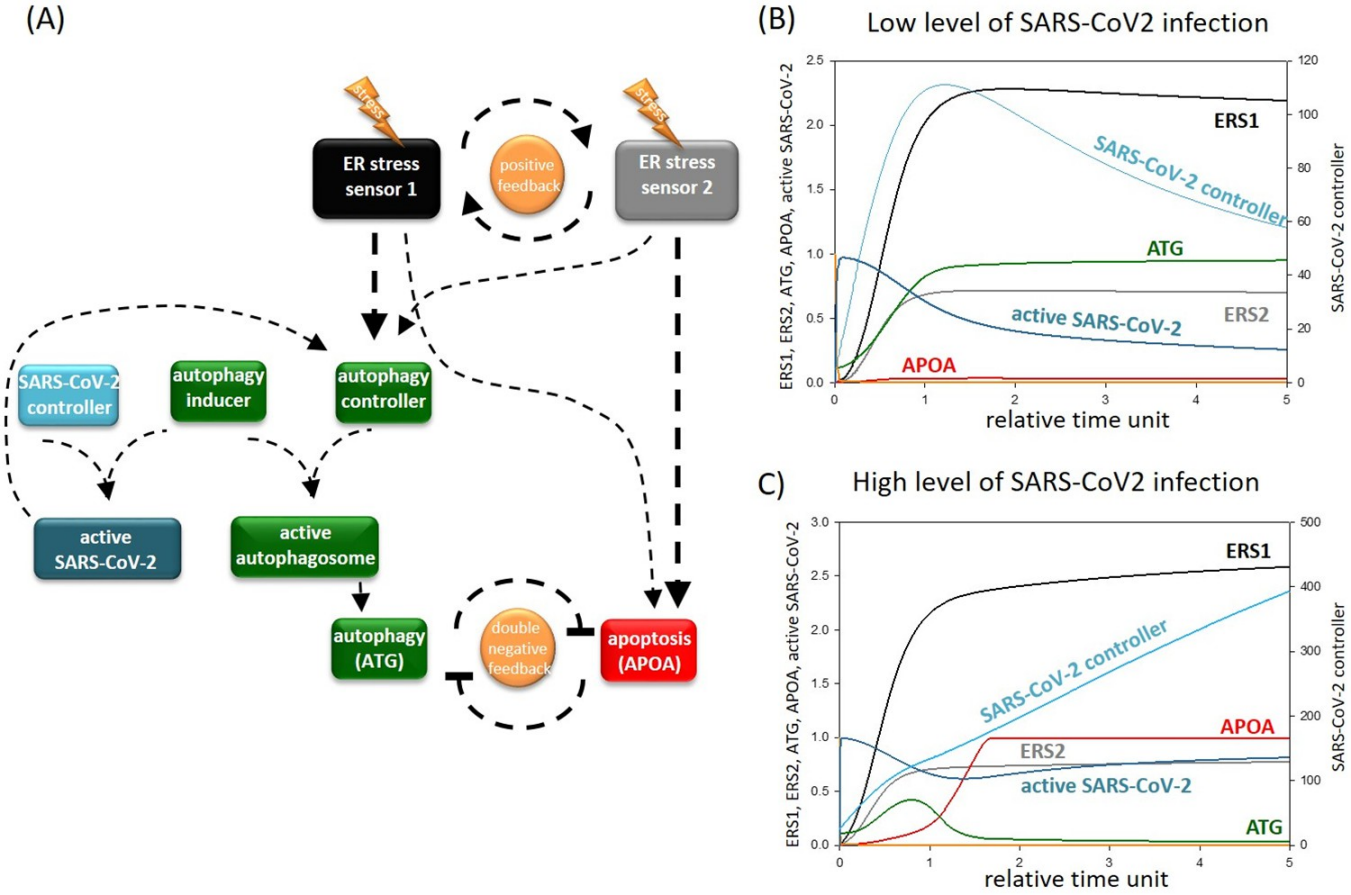
The simple network of autophagy-dependent survival in the presence of SARS-CoV-2. **(A) The wiring diagram of the response mechanism**. The active form of the ER stress sensor 1 and 2, autophagy inducer, autophagy controller, active autophagosome, SARS-CoV-2 controllers, active SARS-CoV2, ATG and APOA are grouped together in isolated coloured boxes. ATG represents the active form of autophagy activator complex, while APOA represents the active form of apoptosis. Dashed lines show how the molecules can influence each other. Blocked end lines denote inhibition. **The temporary profile of computational simulation of (B) mild and (C) severe coronavirus infection**. In case of mild infection CVCT (the initial conditions of SARS-CoV-2 controller) is set to 5 and in case of severe infection CVCT is set to 25 (for the code see S1 File). The relative activity of ER stress sensor 1 (ERS1), ER stress sensor 2 (ERS2), SARS-CoV-2 controller, form of active virus, ATG and APOA is plotted.

Following this, we aimed to address the question of whether CQ/HCQ treatment has an effect on cellular survival during SARS-CoV-2 infection. To answer this question, first we tried to analyse the dynamical characteristic of both mild and severe COVID-19 in the host cell. Based on experimental data, first we estimated how virus infection can be built into our mathematical model. Bonam et al. has shown that SARS-CoV-2 replication starts at the ER-Golgi intermediate component, which is linked to autophagosome biogenesis. They claim that the ER stress response mechanism induced autophagy is significantly affected during virus infection [20]. Previous scientific reports have also shown that SARS-CoV-2 usurped the components of the autophagy pathway [16]. However, the active virus is able to promote autophagy to help the synthesis of the other viruses [62]. According to these experimental data we assume here a so-called SARS-CoV-2 controller which can form a complex with the autophagy inducer resulting in the formation of an active virus in our model. Besides active SARS-CoV-2 promotes the activation of autophagy controller (see Fig 3A). In mild or severe coronavirus infections we supposed that various initial levels of SARS-CoV-2 viruses (i.e. low or high levels) attack the host cell. We assume that the total amount of the autophagy inducer is fixed, and both autophagy and SARS-CoV-2 controllers are needed for their function. Therefore, the severity of the infection seriously determines the competition between autophagy and SARS-CoV-2 controllers and the outcome of SARS-CoV-2 infection.

In the case of low levels of SARS-CoV-2 infection, although some viruses get formed in the host cell, there is an enough amount of free autophagy inducer which can be titrated by the autophagy controller (Fig 3B). Autophagosomes can quickly destroy the active viruses and therefore overcome the infection before the viruses are able to leave the host cell. However, in the case of severe COVID-19 infection, the high amount of SARS-CoV-2 in the host cell do not let work autophagy properly. All the autophagy inducers will form a complex with SARS-CoV-2 controller, and no effective autophagy is observed. The large number of viruses can then leave the host cell and infect the surrounding cells meanwhile the host cell commits apoptotic suicide (Fig 3C).

Thus, our results suggest that mild coronavirus infection can be easily defeated in the host cell by autophagy-dependent survival. However, severe SARS-CoV-2 infection results in apoptotic cell death, enabling high levels of active viruses to escape the host cell and rapidly infect surrounding cells.

### The effect of CQ/HCQ-dependent autophagy inhibition in SARS-CoV-2 infection

**Fig 4:**
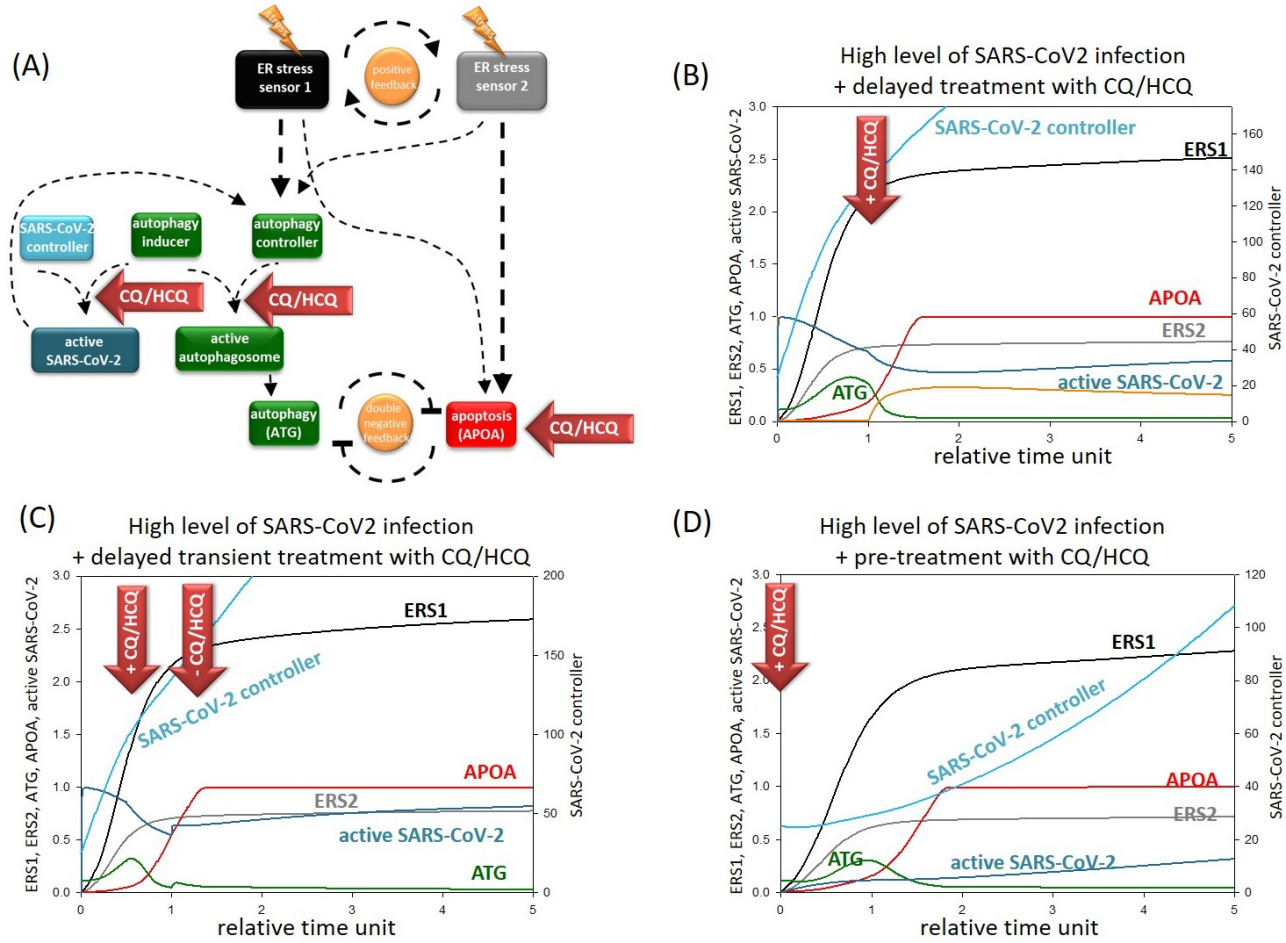
The effect of CQ/HCQ treatment upon severe coronavirus infection. **(A) The wiring diagram of the response mechanism with CQ/HCQ treatment in the presence of SARS-CoV2**. The active form of the ER stress sensor 1 and 2, autophagy inducer, autophagy controller, active autophagosome, SARS-CoV-2 controllers, active SARS-CoV2, ATG and APOA are grouped together in isolated coloured boxes. ATG represents the active form of autophagy activator complex, while APOA represents the active form of apoptosis. Dashed lines show how the molecules can influence each other. Blocked end lines denote inhibition. Red arrows labelled with “chloroquine” show where the drug treatment affects the control network, while yellow “thunders” symbolize the cellular stress. **The temporary profile of computational simulation of delayed (B) permanent or (C) transient treatment with CQ/HCQ or (D) pre-treatment with permanent CQ/HCQ upon severe coronavirus infection**. In case severe infection CVCT (the initial conditions of SARS-CoV-2 controller) is set to 25, while chloroquine treatment was induced by setting CHL = 0.0001 (for the code, see S1 File). The relative activity of ER stress sensor 1 (ERS1), ER stress sensor 2 (ERS2), SARS-CoV-2 controller, form of active virus, ATG and APOA is plotted.

Our next goal was to determine the dynamical characteristic of CQ/HCQ treatment during coronavirus infection. Since we assume here that CQ/HCQ disrupts the autophagosome formation *via* down-regulation of the autophagy controller, both autophagosome and active virus formations are blocked (Fig 4A).

While CQ/HCQ was generally used against severe COVID [20], we first assumed high SARS-CoV-2 infection with high levels of viral agents in the host cell. Since the patients should be treated with extreme caution with CQ/HCQ, high levels of SARS-CoV-2 infection was induced in the cell, then CQ/HCQ was added for a short period (see the red arrows on Fig 4B) and the relative activity of the key elements were followed by computer simulations. Although CQ/HCQ tried to reduce the level of viruses, as the treatment was short-lived, levels of the virus quickly increased. Furthermore, CQ/HCQ not only blocked autophagy, but also induced ER stress, therefore apoptotic cell death killed the host cell.

Since transient CQ/HCQ treatment could not ovecome severe SARS-CoV-2 infection, then “permanent” (or chronic) CQ/HCQ treatment was induced, and the relative activity of the key components of our model were detected by computer simulations (Fig 4C). CQ/HCQ treatment successfully stopped coronavirus infection and decreased the amount of viruses in the host cell. However, not only the formation of SARS-CoV-2 was blocked in CQ/HCQ treatment, but autophagy-dependent survival was also inhibited. Therefore, the ER stress generated apoptosis easily overwhelmed autophagy dependent survival resulting in apoptotic death of the host cell. Thus, our results show that although the spread of SARS-CoV-2 can be blocked by CQ/HCQ treatment, the long-term usage of these agents could have fatal effects at cellular level.

Here we claim that long-term CQ/HCQ treatment might avoid severe COVID-19, but this treatment always results in apoptotic cell death of host cells.

### Pre- treatment with CQ/HCQ is not effective against COVID-19

To further evaluate the negative effects of CQ/HCQ treatment during coronavirus infection, pre-treatment with CQ/HCQ was also modelled with our theoretical analysis. Namely, pre-treatment with CQ/HCQ was also managed followed by intensive COVID-19 infection (Fig 4D). Since high dose of CQ/HCQ itself could induce apoptotic cell death, therefore for CQ/HCQ treatment pre-treatment a half-dose regime of CQ/HCQ was used. In this case no autophagy activation was observed, while it quickly turned on apoptosis assuming that the fatal consequences of CQ/HCQ treatment could not be avoided.

Our result indicates that pre-treatment with CQ/HCQ cannot be used for prevention of SARS-CoV-2 due to its negative effects on the host cell.

## Conclusions

The COVID-19 pandemic caused by SARS-CoV-2 has turned into a worldwide public health priority. In the last year, hundreds of papers have been published focusing on the development of effective treatments against COVID-19 [63]. In addition, many experimental studies have tried to identify both novel or existing drugs to slow down the pandemic. However, theoretical analyses have been largely overlooked. An excellent example of this scientific problem is the case of CQ and HCQ, two well-known antimalarial drugs [20, 38]. They were quickly started to be used in the clinics as an effective drug for treating SARS-CoV-2 in early 2020. Although CQ/HCQ seems to be an important therapeutical option for several autoimmune diseases, recently it has revealed that these drugs had too many negative side effects to use against COVID-19 [6, 38]. Corresponding to various diseases (such as rheumatism and malaria) the slow pharmacokinetics of CQ/HCQ suggest a long-treatment during SARS-CoV-2 infection, too [64]. While novel scientific results show the importance of CQ/HCQ treatment at an early stage of infection [36], the dynamical characteristic of this treatment has not been studied.

Prompted by the lack of such dynamical analysis, in this theoretical study using a mathematical modelling approach, we demonstrate that a cellular level why CQ/HCQ are not effective therapeutic agents for COVID-19 treatment. Since SARS-CoV-2 induces ER stress, we modified our previous ER stress based model to understand the effect of CQ/HCQ during SARS-CoV-2 infection. In this adapted model, we assume a double negative feedback loop between autophagy and apoptosis by taking into account the crucial steps of effective autophagosome formation (see Fig 2A). First low (i.e. tolerable) and high (i.e. non-tolerable) ER stress levels were induced in the host cell (Fig 2B and 2C). Corresponding to our already published data [51, 53], we further revealed here that cells survived with the help of autophagy-dependent self-degradation at low levels of ER stress, however, severe ER stress resulted in apoptotic cell death, meanwhile autophagy was switched off. According to previous experimental data [59], we confirm here that CQ/HCQ blocks the formation of autophagosomes, thereby disrupting autophagy-dependent survival even at low levels of ER stress. This results in apoptotic cell death of the host cell (Fig 2B and 2C).

With the help of already published scientific results (listed in S1 File) our mathematical model revealed that SARS-CoV-2 influences autophagy-induced survival depending on the seriousness of the viral infection in the host cell. Besides ER stress induction, SARS-CoV-2 has been shown to the biogenesis of autophagosomes [20], therefore we assumed here that the replicated viruses and the autophagy activatory complex compete with each other on the free components of autophagosome resulting that autophagy gets down-regulated due to the high amounts of viruses during COVID-19 (Fig 3A).

Here, for the first time, we suggest that the outcome of SARS-CoV-2 infection directly depends on the activity of autophagy during the infection. If the virus level is not too high, sufficient quantities of properly functioning autophagosomes remain to eliminate the active viruses from the host cell (Fig 3B). Therefore, the host cell can easily survive, and stop the spread of the virus in the body. However, high levels of SARS-CoV-2 completely inhibits autophagy in the host cell resulting that the virus level remains high and can infect the surrounding cells, too (Fig 3C). In this case autophagosomes cannot eliminate the viral components but indirectly helps the replication of SARS-CoV-2 suggesting that autophagy acts like a double-edged sword in SARS-CoV-2 infection.

Following this we addressed how CQ/HCQ treatment can affect autophagy induction during SARS-CoV-2 infection in the host cell. At mild coronavirus infection autophagy had a crucial role in digesting and removing the viral components from the cell. Therefore, CQ/HCQ treatment was not found to be advantageous since its negative effect on autophagy disturbed the auto-healing mechanism of the host cell.

CQ/HCQ was quickly introduced to treat people suffering from severe COVID-19 [6, 65], however its exact effects have not yet been published. Our computer simulations have revealed that permanent and transient treatment with CQ/HCQ could affect the level of SARS-CoV-2 in the host cell (Fig 4). Both types of CQ/HCQ addition is able to block autophagosome formation resulting in the down-regulation of virus replication in the host cell. However, the transient addition of CQ/HCQ could not overcome SARS-CoV-2 infection and the levels of the newly formed viruses increased. Meanwhile “permanent” CQ/HCQ treatment completely blocked the virus formation in the host cell suggesting that this type of treatment might have therapeutical value. However, we demonstrate that even the use of “permanent” CQ/HCQ is not beneficial for the host cell, since both CQ/HCQ treatment resulted in the death of host cell via apoptosis induction (Fig 4). We claim that although long-term CQ/HCQ treatment might be effective against SARS-CoV-2, but it can also have a serious negative effect on cellular systems, including killing host cells.

Although our computational analysis is adequately grounded on experimental results, it may have several limitations that would be important to consider. For example, scientific data are being generated from various cellular systems, using several human cell lines with slightly different experimental conditions following SARS-CoV-2 infection etc. Therefore, our predictions need further experimental clarification in the near future.

Theoretical systems biology approaches have been largely neglected in studying SARS-CoV-2 infection. Despite, these systems biology methods are very useful for combining both the molecular and theoretical biological techniques to understand the dynamic characteristics of cellular response mechanisms upon various external or internal signals. We suggest that this type of analysis when theoretical methods precede the experimental study, may allow to decrease the cost of studies. It could also speed up the identification of effective drugs when there is an urgent need for understanding the characteristics of COVID-19 caused by SARS-CoV-2.

## Supporting information

S1 File (DOCX)
