# Supplementary material for: Chloroquine and COVID-19—A systems biology model uncovers the drug’s detrimental effect on autophagy and explains its failure
Source: PLoS One. 2022 Apr 7;17(4):e0266337. doi: 10.1371/journal.pone.0266337 (PMC8989232; doi:10.1371/journal.pone.0266337)
Supplement: S1 File — (DOCX) [file pone.0266337.s001.docx]

Supplementary Material

**“Chloroquine and COVID-19 – a systems biology model uncovers the drug’s detrimental effect on autophagy and explains its failure”**

**S1 Text. Detailed description about the mathematical modelling**

*Basics to build up mathematical models*

A biological regulatory network can be translated into a set of ordinary differential equation (ODE) to describe how the concentration/activity of each control element in the network changes with the time. A generic differential equation depicting the temporal changes of a regulatory element is composed of two parts: production and consumption terms. In a cellular protein-protein regulatory network the production can be given by protein synthesis (i.e. transcription and translation) and/or an activation (i.e. post-translational modification) term, while the consumption can be given by protein degradation and/or inactivation term. Usually synthesis and degradation reactions are described by mass action kinetics, whereas protein activity can be described either by mass action or Michaelis-Menten kinetics. Solving a set of non-linear ODEs gives the time evolution of the relative protein concentration/activity (time courses).

The temporal profiles were computed numerically using *XPP-AUT*. All the simulations presented in the text are based on the following XPP codes which contains ODEs. The rate constants (k) have the dimension of min^-1^ and Michaelis constants (*J*) are dimensionless. The proteins levels/activities are given in arbitrary units (a.u).

*The description of the elements of the theoretical models*

|  | description |
| --- | --- |
| ERS1 | the active form of ER stress sensor 1 |
| ERS2 | the active form of ER stress sensor 2 |
| AUCT | the total level of autophagy executor |
| CVCT | the total level of SARS-CoV-2 |
| DIM1 | the total level of the complex of active autophagy controller and inducer |
| DIM2 | the total level of the complex of active SARS-CoV-2 and autophagy inducer |
| APOA | the active apoptosis inducer |
| AUIT | the total level of autophagy inducer |
| ATG | the active autophagy activator complex |
| S | the level of cellular stress |
| CHL | the level of chloroquine/hydroxy-chloroquine |

*The description of the constants of the theoretical models*

|  | description |
| --- | --- |
| kaers1' | stress-dependent activation of ERS1 |
| kaers1" | ERS2-dependent activation of ERS1 |
| kiers1 | bakcground inctivation of ERS1 |
| ERS1T | the total amount of ERS1 |
| kaers2' | stress-dependent activation of ERS2 |
| kaers2" | ERS1-dependent activation of ERS2 |
| kiers2 | bakcground inctivation of ERS2 |
| ERS2T | the total amount of ERS2 |
| Jap | Michaelis-constant of APOA |
| kaap' | ERS1-dependent activation of APOA |
| kaap" | ERS2-dependent activation of APOA |
| kaap'" | CHL-dependent activation of APOA |
| kiap | background inactivation of APOA |
| kiap' | ATG-dependent inactivation of APOA |
| APOAT | the total amount of APOA |
| ksauc | background activation of AUC |
| ksauc' | ERS1-dependent activation of AUCT |
| ksauc" | ERS2-dependent activation of AUCT |
| ksauc'" | Dim2-dependent activation of AUCT |
| kdauc | background inactivation of AUCT |
| kdauc' | APOA-dependent inactivation of AUCT |
| ATGT | the total amount of ATG |
| kase1 | the complex formation of AUCT-Dim1 and AUIT-Dim1-Dim2 |
| kdse1 | the dissocation of AUCT-Dim1 and AUIT-Dim1-Dim2 |
| kscvc' | Dim2-dependent activation of CVCT |
| kdcvc | backgorund inactivation of CVCT |
| kdcvc' | ATG-dependent inactivation of CVCT |
| kase2 | the complex formation of CVCT-Dim2 and AUIT-Dim1-Dim2 |
| kdse2 | the dissocation of CVCT-Dim2 and AUIT-Dim1-Dim2 |
| Jau | Michaelis-constant of ATG |
| kaatg | background activation of ATG |
| kaatg' | Dim1-dependent formation of ATG |
| kiatg | bakcground inactivation of ATG |
| kiatg' | APOA-dependent inactivation of ATG |

**S2 Text. The codes for computer simulations**

*The code for simulating signal response curve of autophagy*

# an .ode file to simulate the stress-dependent activation of autophagy

# initial conditions

ATG=0, S=0

# differential equations

# ATG represents the active form of autophagy activator complex

ATG' = (kaatg + kaatg'*Dim)*(ATGT-ATG)/(Jau + ATGT-ATG) - (kiatg + kiatg'*APOA)*ATG/(Jau + ATG)

# S represents the stress level

S' = 0

# steady state functions

# ERS1 represents ER stress sensor 1

ERS1 = (kaers1'*S + kaers1"*ERS2)*ERS1T/(kaers1'*S + kaers1"*ERS2 + kiers1)

# ERS2 represents ER stress sensor 2

ERS2 = (kaers2'*S + kaers2"*ERS1)*ERS2T/(kaers2'*S + kaers2"*ERS1 + kiers2)

# APOA represents the active form of apoptosis

APOA = APOAT*GK(kaap'*ERS1 + kaap"*ERS2 + kaap'"*CHL, kiap + kiap'*ATG,Jap,Jap)

# AUCT represents the total form of autophagy controller

AUCT = (ksauc + ksauc'*ERS1 + ksauc"*ERS2)/(kdauc + kdauc'*APOA)

# the complex formation of autophagy controller and autophagy inducer

BB = AUCT + AUIT + kdse1/kase1/CHL

Dim = 2*AUCT*AUIT/(BB + sqrt(BB^2 - 4*AUCT*AUIT))

# 'Goldbeter-Koshand' function (GK)

GB(arg1,arg2,arg3,arg4) = arg2-arg1+arg2*arg3+arg1*arg4

GK(arg1,arg2,arg3,arg4) = 2*arg1*arg4/(GB(arg1,arg2,arg3,arg4)+sqrt(GB(arg1,arg2,arg3,arg4)^2-4*(arg2-arg1)*arg1*arg4))

# parameters

p kaers1'=1, kaers1"=3, kiers1=1, ERS1T=3

p kaers2'=0.5, kaers2"=5, kiers2=5, ERS2T=1

p APOAT=1, Jap=0.02, kaap'=1, kaap"=0.5, kaap'"=0.2, kiap=0.75, kiap'=4

p AUIT=1

p ksauc=0.001, ksauc'=7.5, ksauc"=25, kdauc=0.2, kdauc'=0.75, kase1=500, kdse1=1

p ATGT=1, Jau=0.75, kaatg=0.05, kaatg'=25, kiatg=0.25, kiatg'=75

p CHL=1

#numerics

@ TOTAL=1, METH=stiff, XLO=0, XHI=1, YLO=0, YHI=1, BOUND=100

done

*The code for simulating signal response curve of apoptosis*

# an .ode file to simulate the stress-dependent activation of apoptosis

# initial conditions

APOA=0, S=0

# differential equations

# APOA represents the active form of apoptosis

APOA’ = (kaap'*ERS1 + kaap"*ERS2 + kaap'"*CHL)/(Jap + APOAT-APOA) – (kiatg + kiatg'*APOA)*ATG)/(Jap + APOA)

# S represents the stress level

S' = 0

# steady state functions

# ERS1 represents ER stress sensor 1

ERS1 = (kaers1'*S + kaers1"*ERS2)*ERS1T/(kaers1'*S + kaers1"*ERS2 + kiers1)

# ERS2 represents ER stress sensor 2

ERS2 = (kaers2'*S + kaers2"*ERS1)*ERS2T/(kaers2'*S + kaers2"*ERS1 + kiers2)

# ATG represents the active form of autophagy activator complex

ATG = ATGT*GK(kaatg + kaatg'*Dim, kiatg + kiatg'*APOA,Jau,Jau)

# AUCT represents the total form of autophagy controller

AUCT = (ksauc + ksauc'*ERS1 + ksauc"*ERS2)/(kdauc + kdauc'*APOA)

# the complex formation of autophagy controller and autophagy inducer

BB = AUCT + AUIT + kdse1/kase1/CHL

Dim = 2*AUCT*AUIT/(BB + sqrt(BB^2 - 4*AUCT*AUIT))

# 'Goldbeter-Koshand' function (GK)

GB(arg1,arg2,arg3,arg4) = arg2-arg1+arg2*arg3+arg1*arg4

GK(arg1,arg2,arg3,arg4) = 2*arg1*arg4/(GB(arg1,arg2,arg3,arg4)+sqrt(GB(arg1,arg2,arg3,arg4)^2-4*(arg2-arg1)*arg1*arg4))

# parameters

p kaers1'=1, kaers1"=3, kiers1=1, ERS1T=3

p kaers2'=0.5, kaers2"=5, kiers2=5, ERS2T=1

p APOAT=1, Jap=0.02, kaap'=1, kaap"=0.5, kaap'"=0.2, kiap=0.75, kiap'=4

p AUIT=1

p ksauc=0.001, ksauc'=7.5, ksauc"=25, kdauc=0.2, kdauc'=0.75, kase1=500, kdse1=1

p ATGT=1, Jau=0.75, kaatg=0.05, kaatg'=25, kiatg=0.25, kiatg'=75

p CHL=1

#numerics

@ TOTAL=1, METH=stiff, XLO=0, XHI=1, YLO=0, YHI=1, BOUND=100

done

*The code for time course simulations*

# an .ode file to simulate CHL treatment during various cellular stress

# initial conditions

ERS1=0.0000, ERS2=0.0000, APOA=0.0000, ATG=0.1087, AUC1=0.0003, CVCT=0.0000, DIM1=0.2642, DIM2=7355

# differential equations

# ERS1 represents ER stress sensor 1

ERS1' = (kaers1'*S + kaers1"*ERS2)*(ERS1T-ERS1) - kiers1*ERS1

# ERS2 represents ER stress sensor 2

ERS2' = (kaers2'*S + kaers2"*ERS1)*(ERS2T-ERS2) - kiers2*ERS2

# AUCT represents the total form of autophagy controller

AUCT' = ksauc + ksauc'*ERS1 + ksauc"*ERS2 + ksauc'"*Dim2 - (kdauc + kdauc'*APOA)*AUCT

# CVCT represents the total form of SARS-CoV-2 controller

CVCT' = kscvc'*Dim2 - (kdcvc + kdcvc'*ATG)*CVCT

# Dim1 represents the complex form of autophagy controller and inducer

Dim1' = kase1*CHL*(AUCT-Dim1)*(AUIT-Dim1-Dim2) - kdse1*Dim1 - (kdauc1 + kdauc1'*APOA)*Dim1

# Dim2 represents the complex form of SARS-CoV-2 controller and inducer (active SARS-CoV-2)

Dim2' = kase2*CHL*(CVCT-Dim2)*(AUIT-Dim1-Dim2) - kdse2*Dim2 - (kdauc2 + kdauc2'*ATG)*Dim2

# ATG represents the active form of autophagy activator complex

ATG' = (kaatg + kaatg'*Dim1)*(ATGT-ATG)/(Jau + ATGT-ATG) - (kiatg + kiatg'*APOA)*ATG/(Jau + ATG)

# APOA represents the active form of apoptosis

APOA’ = (kaap'*ERS1 + kaap"*ERS2 + kaap'"*CHL)/(Jap + APOAT-APOA) – (kiatg + kiatg'*APOA)*ATG)/(Jap + APOA)

# parameters

# simulating low SARS-CoV-2 infection: initial conditions of CVCT=5

# simulating high SARS-CoV-2 infection: initial conditions of CVCT=25

p kaers1'=1, kaers1"=3, kiers1=1, ERS1T=3

p kaers2'=0.5, kaers2"=5, kiers2=5, ERS2T=1

p APOAT=1, Jap=0.02, kaap'=1, kaap"=0.5, kaap'"=0.2, kiap=0.75, kiap'=4

p AUIT=1

p ksauc=0.001, ksauc'=3.5, ksauc"=25, ksauc'"”=1, kdauc=3, kdauc'=1, kase1=500, kdse1=1

p kscvc'=175, kdcvc=0.2, kdcvc'=0.75, kase2=10, kdse2=1

p ATGT=1, Jau=0.75, kaatg=0.05, kaatg'=25, kiatg=0.25, kiatg'=75

p CHL=1, S=0

#numerics

@ TOTAL=1, METH=stiff, XLO=0, XHI=1, YLO=0, YHI=1, BOUND=100

done

**S3 Table. The experimental evidences of the regulatory connections to build up the simple wiring diagram of our control network**
